# The Properties of Different Starches under the Influence of Glucono-Delta-Lactone at Different Concentrations

**DOI:** 10.3390/foods12091770

**Published:** 2023-04-25

**Authors:** Afirah Mohd Shukri, Lai-Hoong Cheng

**Affiliations:** Food Technology Division, School of Industrial Technology, Universiti Sains Malaysia, USM, Gelugor 11800, Penang, Malaysia

**Keywords:** starch, green-labelled ingredient, glucono-delta-lactone, acidification

## Abstract

In this study, glucono-delta-lactone (GDL), which is Generally Recognized as Safe (GRAS), was added to native starches to modify their physicochemical properties. The effects of GDL on the molecular weight, pasting properties, flow behavior, gel syneresis, and crystallization properties of potato, tapioca, and corn starches were investigated. GPC results showed that as the GDL concentration increased, the molecular weight of amylose increased, whereas that of amylopectin decreased. An analysis using the Rapid Visco Analyzer revealed that the addition of GDL improved the pasting properties of potato starch, with reduced peak viscosity and breakdown viscosity, and it also improved setback viscosity. On the other hand, tapioca starch degraded substantially after GDL addition, indicating a lower tendency for short-term retrogradation, as reflected in the lower setback viscosity. The effects of GDL on corn starch pasting properties were very similar to those observed for tapioca starch, but the changes were relatively subtle. In terms of flow behavior, GDL addition decreased and increased the flow index values of the potato and tapioca starch pastes, respectively. However, the effect of GDL addition on the flow index value of the corn starch paste was found to be insignificant. The results also showed that the percentage of syneresis under the influence of GDL depended on the starch botanical origin—that is, potato starch, 14–18%, tapioca starch, 10–13%, and corn starch, 17–20%—which was substantiated by crystallinity analysis. It was observed that GDL has the potential to be used for starch modification because it creates desirable functionalities with the advantage of being a green-labelled ingredient.

## 1. Introduction

In recent years, plant-based foods have grown in popularity, especially as a result of vegans (no animal-based products at all), vegetarians (no meat, but some dairy and eggs allowed), pescatarians (some fish allowed, but no meat), and flexitarians (a reduced meat diet). People are becoming increasingly concerned about the health, ethical, environmental, and sustainability issues associated with animal-derived industries [1]. Consumers’ eating behaviors in regard to plant-based diets are changing because of the belief that plant products are healthier than animal-based foods, which contain saturated fat and cholesterol, and are linked to a high prevalence of some chronic diseases such as cardiovascular disease [2,3,4]. Furthermore, some people switch to plant-based diets because of ethical concerns, such as the killing and slaughtering of animals [5]. In addition, livestock rearing has a greater impact on pollution, greenhouse gas emissions, water use, and habitat and biodiversity loss than planting plants for food [6,7]. To achieve the UN Sustainable Development Goals (SDGS) for sustainable consumption and production (SDG#12), the food production system must be changed. This trend has motivated the food industry to venture into plant-based products, such as meat, milk, eggs, cheese, and yoghurt analogs that mimic the physicochemical, sensory, or nutritional attributes of the original animal-based versions to lessen the burden of environmental impacts, and reduce health issues caused by animal-derived food [8].

Because the molecular and physicochemical properties of plant-derived ingredients differ significantly from those of animal-derived ingredients, it is critical to understand the fundamental attributes of plant-derived ingredients and how they can be assembled into structures that resemble those found in animal products [8]. Starch, a complex carbohydrate, is an important functional ingredient that can be used to assemble analogs of animal products. In plant-based foods, starch acts as a bulk ingredient, providing structure and texture to the food and is a source of energy [9]. It is also used to provide a variety of functional attributes in plant-based foods, such as sweetness, thickness, gelling, emulsification, structure formation, stabilization, and fluid holding, or can be used as a filler, helping to improve the texture, consistency, and shelf-life of products [10]. Moreover, it can be sourced from various plants, including corn, potato, tapioca, rice, wheat, and barley [11]. It is also cost effective and sustainable. As modified starch has better functionalities than native starch, it is frequently used in plant-based products [12]. However, conventional methods of starch modification require excessive reagents and may cause environmental contamination from unreacted chemicals [13]. In a study that determined plant-based food trends from a business perspective, Aschemann-Witzel et al. [14] found that more innovations in plant-based products that are healthy, safe, and clean are required for the plant-based food sectors to grow on a larger scale.

Thus, the purpose of this study was to investigate the effects of glucono-delta-lactone (GDL), a weak acid which is a green-labelled ingredient with a Generally Recognized as Safe (GRAS) status, on the properties of selected native starches. GDL is an acidity regulator that controls the pH of foods and can be used as a preservative. It is also used as a curing and pickling agent, a leavening agent, and a sequestrant [15]. Based on a study by Sumitra et al. [16], GDL dissolves gradually and produces an initial sweet taste before transitioning to a mildly acidic taste when it reaches equilibrium pH. When compared to other organic acids such as fumaric acid, tartaric acid, malic acid, acetic acid, succinic acid, lactic acid, and ascorbic acid, GDL yields the least tartness. Therefore, it is commonly used as a coagulant in the production of tofu and cheese, as well as a preservative in meat products, pasta, noodles, and bakery products. The use of GDL as an acidifying agent can reduce the pH of food to less than 4.6, requiring only pasteurization to ensure microbial safety. This is advantageous because a milder manufacturing process results in better nutrient retention and less toxic compound formation [17,18].

Despite its widespread use, research on the effects of GDL on the physicochemical properties of native starch has not yet been reported. Kim et al. [19] reported that the hardness of cooked rice increased significantly after being acidified with GDL. In addition, GDL has been added to udon (a wheat-based noodle) flour premix to lower its pH and produce fresh udon that can be marketed as a shelf-stable product under ambient conditions [16]. On the other hand, Low et al. [20] attempted to dip fresh rice noodles into GDL solution followed by in-pack pasteurization. GDL-treated noodles were reported to show enhanced storage stability and were not sticky or fragile during storage.

Hence, the objective of this study was to investigate and compare the effects of GDL on the pasting properties and molecular weight changes of potato starch, tapioca starch, and corn starch, as well as the flow behavior of the paste formed therefrom. In addition, the influence of GDL on gel syneresis and the crystallinity of the starches was studied. 

## 2. Materials and Methods

### 2.1. Materials

Potato starch (15% *w*/*w* moisture, 0.2% *w*/*w* protein, 0.41% *w*/*w* fat, 21% amylose), tapioca starch (13.5% *w*/*w* moisture, 0.15% *w*/*w* protein, 0.56% *w*/*w* fat, 16% amylose), corn starch (12.5% *w*/*w* moisture, 0.38% *w*/*w* protein, 0.97% *w*/*w* fat, 22% amylose), and food grade glucono-delta-lactone (GDL) were purchased from Euro Chemo Pharma Sdn. Bhd., Penang, Malaysia. The composition of the individual starches was analyzed in our laboratory. 

### 2.2. Preparation of GDL Solution

A food grade GDL solution was prepared at different concentrations (0.00%, 0.05%, 0.10%, and 0.50% *v*/*w*) and it was left overnight before use. The pH values of distilled water and GDL solutions at 0.00%, 0.05%, 0.10%, and 0.50% were 6.70, 3.77, 3.55, and 3.12, respectively. These values were measured using a pH meter (Eutech Instruments pH 510, Singapore). 

### 2.3. Pasting Analysis

The effects of different GDL concentrations on the pasting properties of the different starch samples were evaluated using a Rapid Visco Analyzer (RVA, Series 4, Newport Scientific Pty. Ltd., Warriewood, Australia). The generated data were analyzed using the RVA software program (Thermocline for Windows, Version 2.2 TCW). The moisture content of the starch samples was found to be 14%. For each run, starch (2.5 g) was weighed into an RVA canister, followed by the addition of different concentrations of the GDL solution (0.00%, 0.05%, 0.10%, and 0.50%) to prepare for a 10% starch suspension. The starch suspension was analyzed under continuous shear conditions. The suspension was vigorously sheared at 960 rpm, heated from 25 °C to 90 °C in 3 min 42 s, held at 90 °C for 4 min 30 s, before cooling to 50 °C in 3 min 48 s. The RVA attributes recorded were peak viscosity, peak time, trough viscosity, pasting temperature, breakdown viscosity, final viscosity, and setback viscosity. The starch pastes prepared from the RVA were subjected to pH measurement and flow analysis.

### 2.4. Starch Paste pH Values Determination

The freshly pasted sample from the RVA was transferred into a beaker and blended with an equal amount of distilled water. The pH value was measured using a pH meter (Eutech Instruments pH 510, Singapore).

### 2.5. Flow Analysis

The steady-state flow analysis was conducted using a Rheometer AR-1000N and the fresh starch pastes were produced by RVA. All measurements were conducted at 50 °C and a 40 mm diameter parallel plate geometry was used. The starch paste was placed between the geometry and the Peltier plate with a 1 mm gap. The flow experiments were conducted under steady-shear conditions with 0.1 to 200 s^−1^ shear rate. According to the “Best Fit” routine, it was found that the best fit model for the data is the Herschel–Bulkley model with a standard error value below 10 and a regression coefficient (R^2^) value of more than 0.99. The Herschel–Bulkley equation is

σ = σ_ο_ + *K*γ*^n^*

where σ is the shear stress (Pa), γ is the shear rate (s^−1^), *K* is the consistency coefficient (Pa s*^n^*), *n* is the flow behavior index (dimensionless), and σ_ο_ is the yield stress (Pa). 

### 2.6. Starch Gel Syneresis Analysis

The syneresis tendencies of all starch samples were determined according to the method described in Singh et al.’s study [21]. The starch suspension (2%, *w*/*v*) was heated at 85 °C in a 50 mL falcon centrifuge tube for 30 min in a water bath, followed by rapid cooling in an ice-water bath. The starch gel samples prepared were stored for 24, 48, and 120 h at 4 °C. The syneresis was measured as the percentage of water released after the process of centrifugation at 3000 rpm for 15 min. 

### 2.7. Crystallinity Changes Observation by X-ray Diffractometry (XRD)

The starch gel samples stored for 48 h (from Section 2.6) were freeze-dried and subjected to a long-range ordered structure investigation by an X-ray diffractometer (D5000, SIEMENS, Karlsruhe, Germany) operating at 40 mA and 40 kV with Cu-Kα radiation (k = 1.5406 Ǻ). The samples were equilibrated at ambient conditions overnight prior to analysis. The XRD patterns were obtained from 4° to 40° with a step size of 0.02°. The degree of crystallinity was estimated in a quantitatively manner following the method of Nara and Komiy [22] using Diffrac Plus V4 software.

### 2.8. Gel Permeation Chromatography

Freeze-dried starch powder from Section 2.7 was subjected to gel permeation chromatography. The weight average molecular weight (M_w_), polydispersity (M_w_/M_n_), and intrinsic viscosity (IV, η) of non-GDL- and GDL-treated starches were determined using a Malvern Viscotek Triple Detection GPC Max (Light Scattering Detector, RI Detector and Viscometer) Gel Chromatography (GPC) system, which consists of an inline eluent degasser, a syringe-loading sample injector equipped with a 100 uL sample loop, and a dual linear (connected in series) Viscotek A6000M Aqueous GPC/SEC column packed with hydroxy methacrylate polymer, following the method of Chong et al. [23]. The mobile phase was an aqueous solution of 0.1 mol equivalent/L NaNo_3_ and 0.02% sodium azide at a flow rate of 1.0 mL/min. The column was calibrated using dextran of known molecular weight. Starch samples (0.1 g) were prepared in dimethyl sulphoxide (DMSO), shaken for an hour in a 95 °C water bath. The sample was then stirred at room temperature for 24 h. A 1.5 mL aliquot was taken, and 6 mL of absolute alcohol was added. The precipitated starch was recovered by centrifugation at 2000× *g* for 30 min and washed twice with ethanol by centrifugation for 15 min to remove DMSO. The precipitated starch was then redissolved in 5 mL of deionized water and stirred for 30 min in a heated water bath. The mixture was then centrifuged at 2000× *g* for 15 min to remove insoluble residues prior to injection. The chromatograms have been resolved into two distinct peaks, namely Fraction I which appears at the early retention time while Fraction II appears later. The M_w_, M_w_/M_n_, and IV were analyzed and calculated using OmniSEC software Version 5.02.438 (Viscotek Corporation, Houston, TX, USA). 

### 2.9. Statistical Analysis

All experiments were conducted in triplicate. A one-way analysis of variance (ANOVA) was used to analyze the significant effects of GDL treatment on each property studied at a 95% confidence level using IBM SPSS Statistics Campus Edition V24.0. Duncan’s test was carried out to study the significant differences between mean values. 

## 3. Results and Discussion

### 3.1. Gel Permeation Chromatography Analysis

Table 1 presents the effects of GDL on weight average molecular weight (M_w_), polydispersity (M_w_/M_n_), and intrinsic viscosity (η) of potato, tapioca, and corn starch. GPC is used to characterize polymers and separate mixtures into discrete fractions. The starch samples were separated into two main fractions: a high molecular weight fraction that eluted at the void volume (Fraction I), and a lower molecular weight fraction that entered the gel and was eluted after (Fraction II). As a result, a comparison of molecular weight distribution can be made according to the retention time of the samples [24]. Thus, Fraction I with a shorter retention time was the amylopectin, and Fraction II was the amylose with a smaller molecular size. It is apparent in Table 1 that the molecular weight of Fraction I decreases as the concentration of GDL increases, in all types of starch sample. This is consistent with the studies conducted by Karim et al. [25] and Abdorreza et al. [26], which found that the molecular weight of starch components, particularly amylopectin, was reduced by acid hydrolysis. According to Majzoobi et al. [27], the proton released from acid dissociated in water can destabilize and depolymerize glycosidic bonds of starch molecules, resulting in smaller molecules because of starch degradation. This explains why the molecular weight of Fraction I has decreased in all the starch samples. On the other hand, Fraction II, which is amylose, increases in molecular weight when higher GDL concentration is added, possibly resulting from the hydrolysis of Fraction I. The hydrolysate from Fraction I could have merged with amylose molecules in Fraction II. These findings suggest that highly branched amylopectin is more prone to degradation compared to the linear amylose chains under acidic conditions. These data also identify that potato starch is highly susceptible to acid hydrolysis compared to tapioca starch and corn starch. 

The polydispersity index (M_w_/M_n_) is a measure of the broadness of a polymer’s molecular weight distribution. The polydispersity index will be higher with a wider molecular weight distribution [28]. The results show that polydispersity index increased with GDL concentration. This indicates that the molecular weight distribution of starch molecules is dependent on GDL concentration. The intrinsic viscosity was primarily determined by the molecular chain length [29]. The acidification process of starch shortens the chains, and, thus, it reduces the molecular weight and the intrinsic viscosity of GDL-treated starch as compared to the native starch. 

### 3.2. Pasting Properties

Table 2 shows the pasting properties of potato, tapioca, and corn starches pasted with GDL solutions at different concentrations. As observed, the addition of GDL to the aqueous medium has generated different pasting effects in all the samples studied. The results showed that when the dispersion medium was changed from distilled water to GDL solution, the pasting time and temperature of potato, tapioca, and corn starch samples did not differ significantly (data not shown). The pasting temperature for potato starch ranged from 69.45 to 68.70 °C, corn starch from 79.80 to 81.45 °C, and tapioca starch from 68.70 to 68.50 °C. On the other hand, the GDL has seriously influenced the peak viscosity, trough viscosity, breakdown viscosity, final viscosity, and setback viscosity of the samples studied. These effects can be attributed to the differences in size, shape, granule organization, chemical and molecular properties, starch composition (amylose/amylopectin ratio), et cetera, among starches of different botanical origins. 

Peak viscosity refers to the maximum swelling capacity of starch granules during heating. The results in Table 2 show that the peak viscosity of potato, tapioca, and corn starch samples was decreased with the progressive increase in the GDL concentration. Native starch without the addition of GDL shows the highest peak viscosity. According to Abera et al. [30], native starch has strong connections among its molecules, resulting in a more stable conformation and a lower tendency for amylose to leach out of granules. The lower viscosity in GDL-treated starches indicates that acid easily attacks the amorphous regions of granules, reducing the molecular mass of amylopectin and causing starch granule disintegration (Putri et al.) [31]. This result is in line with the GPC results. One could ascribe this phenomenon observed to the inherent phosphate groups found in potato, tapioca, and corn starch [32,33,34]. The phosphate groups were responsible for the swelling capacity of the starch granules during pasting due to charge repulsion. Moreover, the phosphorus type also influences starch performance. Most cereal starches contain phosphorus primarily as lysophospholipids, which tend to complex with the amylose and reduce its water-binding capacity, resulting in lower peak viscosity. The phosphorus in tuber and root starches, such as potato and tapioca, is in the form of phosphate monoesters, which appear as negatively charged groups on the starch molecule. The ionic repulsion generated by these groups weakens the molecules’ association while increasing water-binding capacity and swelling power as well as the peak viscosity [35]. Upon GDL acidification, the swelling capacity could have been suppressed by a charge shielding effect caused by the protons released by the GDL molecules. As a result, the peak viscosity was decreased, indicating that a decreased viscous load occurred when starches were cooked in GDL solution. The effect of reduction in peak viscosity upon GDL acidification declined in the order of potato > corn > tapioca starch. This result trend is in tandem with the findings of some researchers who have reported that the inherent potato starch phosphate content is much higher than in tapioca starch and corn starch [32,33,34].

Trough viscosity is the hot paste viscosity when the starch is fully cooked. GDL acidification is found to reduce the trough viscosity and the effect is particularly higher in tapioca starch and potato starch as compared to corn starch over the GDL concentration range studied. The large drop in trough viscosity may suggest a larger extent of amylopectin damage upon pasting in tapioca and potato starch than corn starch. This observation is substantiated by the results presented in the previous section (Table 1), where a large drop in weight average molecular weight of Fraction I is observed in potato starch, followed by tapioca starch and corn starch. 

According to the data shown in Table 2, when GDL concentration was increased from 0 to 0.5%, the breakdown viscosity of potato starch decreased, while the reverse is true for tapioca and corn starch. A large breakdown value indicates low process tolerance. Since breakdown viscosity is defined as the difference between peak viscosity and trough viscosity, as the two parameters are dropping at different rates, different breakdown viscosity responses were recorded. For potato starch, the peak viscosity was dropping at a faster rate than its corresponding trough viscosity upon GDL acidification. Thus, a decrease in breakdown viscosity was observed. The opposite effect was seen in tapioca and corn starch, wherein the peak viscosity was marginally affected while the trough viscosity drastically dropped with a progressive increase in the GDL concentration. This resulted in an increase in breakdown viscosity in tapioca and corn starch with GDL acidification. When gelatinized starch paste was sheared and added with acid, the glycosidic bonds were hydrolyzed and the starch granules became weaker, consequently collapsed, and dispersed, resulting in increased breakdown viscosity [36].

The final viscosity is the cold viscosity that refers to the ability of starch molecules to form a viscous paste after cooling. The final viscosity result is found to decrease with a progressive increase in the GDL concentration for all samples over the GDL concentration studied, with the extent of reduction following the order of tapioca > potato > corn. This trend suggests that the GDL acidification disrupted the chain–chain association in tapioca starch more than in the other two counterparts. When starch paste is cooled, starch molecules start to reassociate and form a network. The tendency for these chain–chain interactions to happen is reflected from the setback value, calculated as a difference between final viscosity and trough viscosity. The results show that corn starch and tapioca starch setback values were found to have decreased, and the setback value of potato starch increased upon GDL acidification. Thus, it is believed that the corn and tapioca starch molecules could have been degraded but to different extents upon GDL acidification. In this case, corn starch is found to be more resistant to GDL hydrolysis than tapioca starch, and that could probably be due to the high amount of amylose–lipid complex found in cereal starches; meanwhile, tapioca starch is susceptible to GDL depolymerization to short chains until it voids the capability to entangle and reassociate to form a cohesive network. The reverse trend is found for potato starch, wherein GDL acidification promoted chain–chain interactions upon cooling. Both lower final viscosities and setback values of tapioca starch indicate that tapioca starch could offer a potentially higher concentration/dosage application, especially where higher solid content is favored, with low tendency towards retrogradation. 

### 3.3. Starch Pastes’ pH Values

All the starch samples pasted in the GDL solution showed a pH value below 4.6 (Table 2); this is a critical pH that allows foods to be pasteurized to achieve commercial sterility, and be stored without refrigeration due to adequate inhibition of growth and toxin formation from bacteria, especially those causing botulism. It is worth noting that as low as 0.05% GDL solution is sufficient to reduce the pH value of a 10% (*w*/*w*) starch paste to approximately pH 3. Moreover, it is important to note that GDL could be a better acidulant compared to other acid regulators because it provides a lower level of tartness. Sumitra et al. [16] state that when GDL is dissolved in water, it produces a sweet taste initially followed by a tartness. Thus, GDL could be a better option for formulating acidified starch-based food for in-package thermal pasteurization applications. 

### 3.4. Flow Properties

**Figure 1:**
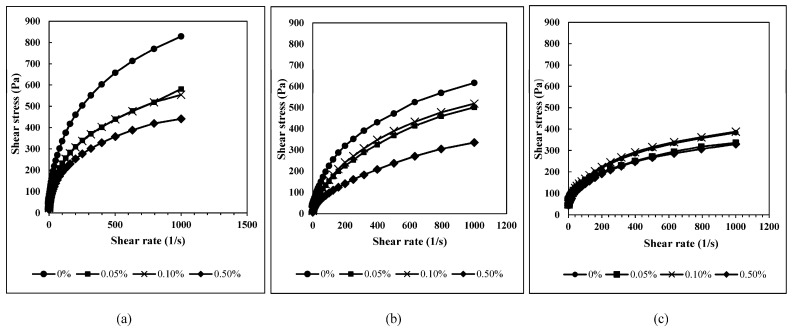
Effects of different GDL concentrations (●—0%; ■—0.05%; X—0.10%, and ♦—0.50%) on flow properties of different types of starch, (**a**) potato starch, (**b**) tapioca starch, and (**c**) corn starch, based on Herschel–Bulkley model.

Figure 1 illustrates the stress versus shear rate plots for all samples studied as a function of GDL concentration. Through a visual inspection, it was noted that GDL influenced the flow properties of potato starch more than tapioca starch, while the least effect was seen in corn starch. All the samples exhibited shear thinning properties, where viscosity decreased with the progressive increase in the shear rate. From the “Best Fit” routine, it was found that the Herschel–Bulkley model was the best fit model, with a standard error value below 10 and a regression coefficient (R^2^) value of more than 0.99. From the model fitted, the yield stress, the consistency coefficient (k), and the flow behavior index (*n*) are calculated and summarized in Table 3. 

Yield stress is the stress value below which the stress will result in no observable flow situation in the material tested [36]. The yield stress values in Table 3 demonstrate that potato starch pastes without GDL acidification showed a very high yield stress value (19.33 Pa). In the presence of GDL (0.05% to 0.5%), the yield stress value dropped drastically to 3.34–2.60 Pa. The opposite was observed for the corn starch, where yield stress increased marginally from 36.33 Pa (0% GDL) to 41.98 Pa (0.5% GDL), and GDL was found not to significantly affect the yield stress value of the tapioca starch paste. This suggests that potato starch paste handling properties were enhanced upon GDL acidification while the reverse is true for corn starch [37,38,39]. The flow index values (*n*) for all the samples studied ranged from 0.352 to 0.563, indicative of a non-Newtonian shear thinning behavior. Interestingly, GDL acidification decreased and increased the flow index value of potato and tapioca starch paste, respectively. The effect of GDL on corn starch paste flow index value was found to be non-significant. This reveals that potato and tapioca starch become more and less shear thinning when being sheared, respectively, upon GDL acidification. Moreover, treatment with GDL acidification led to a lower consistency (*K*) value in potato and tapioca starch, and no significant changes were detected in corn starch paste (*p* < 0.05). This result is in line with the GPC results, where corn starch molecular properties were found to be least affected by GDL when compared to potato starch and tapioca starch.

### 3.5. Gel Syneresis Properties

**Figure 2:**
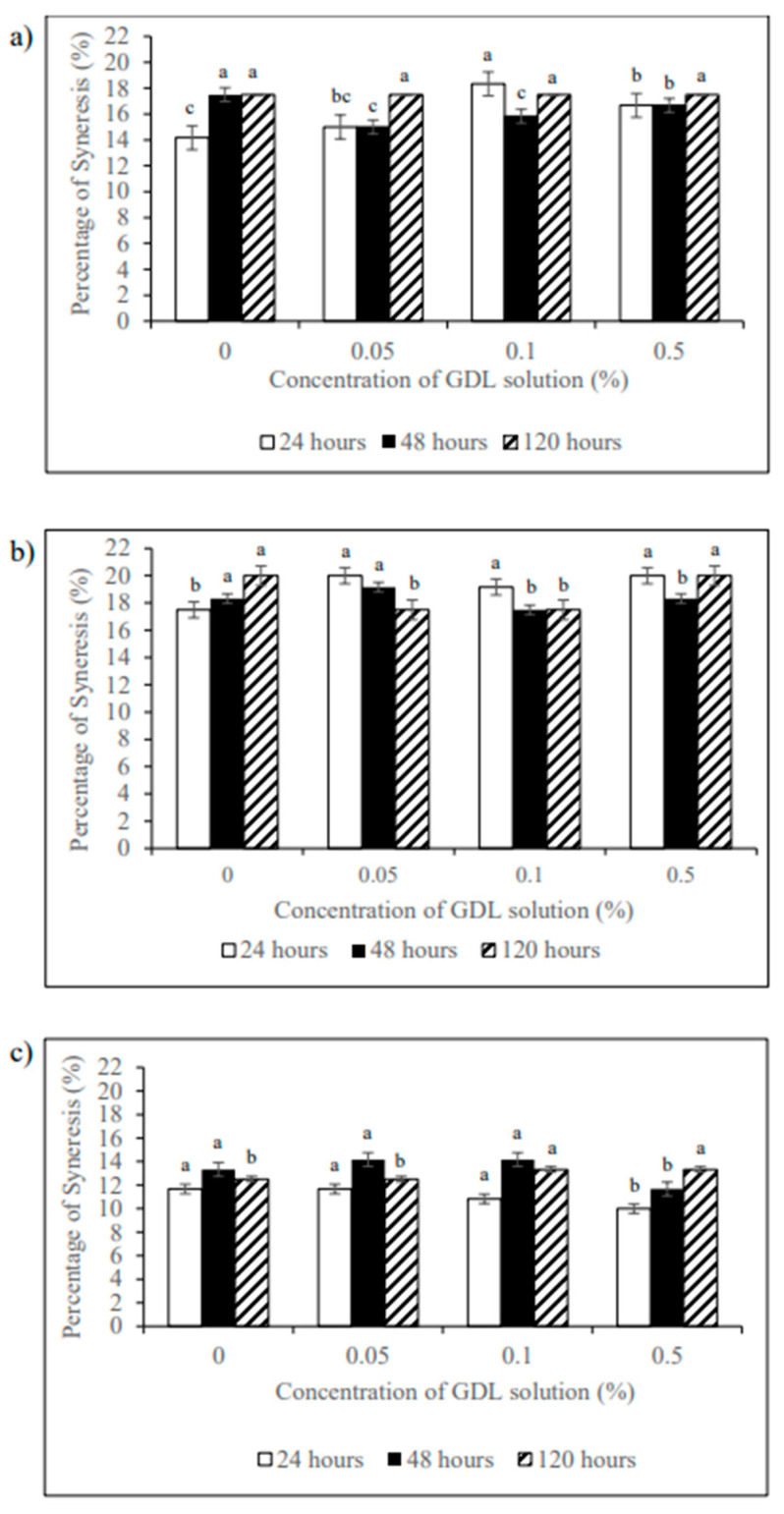
Percentage of syneresis of starch gels: (**a**) potato starch; (**b**) corn starch; (**c**) tapioca starch pasted with different concentrations of GDL solution. Means with different letters were significantly different (*p* < 0.05) regarding GDL concentration within the same storage time.

Starch gel syneresis happens when starch molecules interact and expel liquid from their network. Figure 2 displays the percentages of syneresis shown in potato, tapioca, and corn starch as a function of GDL concentration and storage time at 4 °C. The higher the syneresis values are, the poorer the storage stability is. The GDL-treated samples showed higher syneresis compared to the native starches. The syneresis increased with increasing GDL concentration. The addition of GDL resulted in the formation of amylose, which can reduce the water absorption of the samples. Therefore, the starch added with GDL could not hold as much water, resulting in higher syneresis of the samples [27]. Hence, the effects of GDL acidification on the degree of syneresis were found to be GDL concentration- and storage-time-dependent. The results also showed that the percentage of syneresis that occurred was dependent on the starch type, i.e., potato starch, 14–18%; tapioca starch, 10–13%; and corn starch, 17–20%. In other words, tapioca gel is the most stable during storage in terms of syneresis, followed by potato starch gel and corn starch gel in the presence of GDL. This may be associated with the low amylose content in tapioca starch [40].

### 3.6. Gel Crystalline Properties

**Figure 3:**
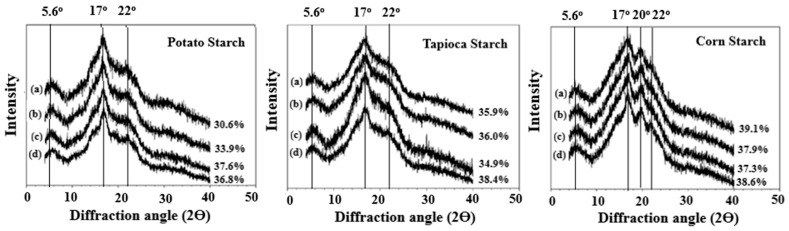
X-ray diffraction spectra of potato, tapioca, and corn starches pasted with different concentrations of GDL: (**a**) 0%; (**b**) 0.05%; (**c**) 0.1%; (**d**) 0.5%.

X-ray diffraction analysis was carried out to analyze the effects of GDL acidification on starch gel crystalline properties (Figure 3). Potato and tapioca starch molecules were found to recrystallize into similar crystalline packing arrangements and to form an X-ray pattern with some characteristic peaks observed at 5.6°, 17°, and 22° 2Ɵ. For corn starch, an additional peak at 20° 2Ɵ was evident. Upon GDL acidification, the potato and tapioca starch samples showed slightly sharper peaks at 2Ɵ = 17° and 22°. This could be attributed to the increase in crystallinity [41], and/or increased perfection in starch chains crystallites [22] that could have resulted from the acid-thinning effects of GDL. The results in Figure 3 show that all the starches studied were found to recrystallize to different degrees of crystallinity upon storage, i.e., potato (30.6–36.8%), tapioca (35.9–38.4%), and corn (39.1–38.6%). The absolute values indicate that corn starch is the most highly crystallized starch upon gelling and the least influenced by GDL concentration, followed by tapioca and potato starch. This observation is in tandem with the pasting properties results from which it can be deduced that susceptibility to GDL-hydrolysis was in the order of potato ≥ tapioca > corn. It has been suggested that starch hydrolysis allowed the reordering of the starch chain segments, and thus resulted in a more crystalline structure with a sharper X-ray pattern [42,43].

## 4. Conclusions

In summary, the molecular weight, pasting properties, flow behavior, gel syneresis, and crystallinity of native starch were altered by the addition of GDL. These effects differed depending on the starch source and GDL concentration. Potato starch produced the most significant results overall. Corn starch was the least sensitive to the addition of GDL, followed by tapioca starch. These effects are attributed to the charged shielding effects caused by the protons released by GDL molecules. The results of the present study may contribute to the generation of new knowledge on predicting and manipulating the quality of a product wherein native starch is used together with GDL as an acidifying agent to reduce the pH of the product. As a result, this may aid in the development of plant-derived food product quality in order to broaden food product options for vegan and vegetarian consumers. Additional studies are still needed to fully evaluate the effect of GDL addition on other types of starch, and their applicability in plant-based foods.

## Figures and Tables

**Table 1:** Gel Permeation Chromatography (GPC) analysis of different types of starch in different GDL concentrations.

Types of Starch	GDL Concentration (%)	Fraction I			Fraction II		
		Molecular Weight (M_w_) (Da) × 10^6^	Polydispersity (M_w_/M_n_)	Intrinsic Viscosity (η, DL/g)	Molecular Weight (M_w_) (Da) × 10^3^	Polydispersity (M_w_/M_n_)	Intrinsic Viscosity (η, dL/g)
Potato	0	5.51 ± 0.07 ^a^	1.55 ± 0.04 ^d^	0.304 ± 0.005 ^a^	6.80 ± 0.91 ^d^	3.06 ± 0.24 ^b^	1.70 ± 0.001 ^a^
	0.05	1.16 ± 0.12 ^b^	6.82 ± 0.16 ^c^	0.392 ± 0.008 ^b^	8.80 ± 0.01 ^c^	3.54 ± 0.59 ^b^	1.63 ± 0.006 ^b^
	0.1	1.16 ± 0.13 ^b^	7.32 ± 0.31 ^b^	0.390 ± 0.060 ^b^	9.83 ± 0.76 ^b^	5.29 ± 0.36 ^a^	1.49 ± 0.009 ^c^
	0.5	0.61 ± 0.05 ^c^	8.84 ± 0.30 ^a^	0.373 ± 0.006 ^c^	10.2 ± 0.74 ^a^	5.50 ± 0.34 ^a^	1.47 ± 0.002 ^c^
Tapioca	0	4.60 ± 0.06 ^a^	6.39 ± 0.32 ^c^	0.674 ± 0.002 ^a^	4.16 ± 1.17 ^d^	4.70 ± 0.22 ^b^	0.678 ± 0.009 ^a^
	0.05	2.55 ± 0.08 ^b^	9.05 ± 0.84 ^b^	0.537 ± 0.002 ^b^	5.23 ± 0.48 ^c^	4.67 ± 0.15 ^b^	0.665 ± 0.004 ^b^
	0.1	2.47 ± 0.04 ^b^	10.01 ± 0.02 ^ab^	0.519 ± 0.040 ^b^	5.50 ± 2.23 ^b^	4.51 ± 0.31 ^b^	0.605 ± 0.003 ^c^
	0.5	1.81 ± 0.06 ^c^	10.50 ± 0.40 ^a^	0.506 ± 0.002 ^b^	6.55 ± 0.25 ^a^	7.99 ± 0.03 ^a^	0.593 ± 0.000 ^d^
Corn	0	3.15 ± 0.17 ^a^	1.99 ± 0.06 ^b^	0.489 ± 0.001 ^a^	3.83 ± 0.15 ^d^	2.86 ± 0.05 ^c^	1.200 ± 0.003 ^a^
	0.05	1.40 ± 0.02 ^b^	2.18 ± 0.15 ^b^	0.154 ± 0.006 ^b^	4.87 ± 0.68 ^c^	4.15 ± 0.37 ^b^	0.744 ± 0.000 ^b^
	0.1	1.36 ± 0.03 ^b^	2.27 ± 0.96 ^b^	0.142 ± 0.003 ^b^	5.15 ± 0.14 ^b^	4.65 ± 0.30 ^b^	0.590 ± 0.005 ^c^
	0.5	1.33 ± 0.07 ^b^	9.91 ± 0.54 ^a^	0.138 ± 0.012 ^b^	6.14 ± 0.69 ^a^	5.94 ± 0.26 ^a^	0.538 ± 0.001 ^d^

Values expressed mean ± standard deviation (*n* = 3). Data of starch samples from the same botanical origin with different superscript letters in the same column were significantly different (*p* < 0.05).

**Table 2:** Pasting characteristics of different types of starches pasted with different concentrations of GDL solutions.

Types of Starches	Concentration of GDL Solution (%)	pH Value	Pasting Parameters *				
			PV (Pa.s)	TV (Pa.s)	BD (Pa.s)	FV (Pa.s)	SB (Pa.s)
	0.00	5.70 ± 0.03 ^a^	8.58 ± 0.09 ^a^	2.80 ± 0.02 ^a^	5.72 ± 0.08 ^a^	3.19 ± 0.05 ^a^	0.38 ± 0.03 ^b^
	0.05	3.11 ± 0.02 ^b^	6.63 ± 0.05 ^b^	2.23 ± 0.05 ^b^	4.40 ± 0.08 ^b^	2.81 ± 0.07 ^b^	0.58 ± 0.02 ^a^
Potato	0.10	3.11 ± 0.20 ^b^	6.06 ± 0.02 ^c^	1.99 ± 0.04 ^c^	4.07 ± 0.05 ^c^	2.58 ± 0.05 ^c^	0.59 ± 0.02 ^a^
	0.50	2.88 ± 0.02 ^c^	5.07 ± 0.07 ^d^	1.39 ± 0.02 ^d^	3.68 ± 0.05 ^d^	1.98 ± 0.05 ^d^	0.59 ± 0.04 ^a^
	0.00	5.85 ± 0.03 ^a^	3.45 ± 0.01 ^a^	1.61 ± 0.03 ^a^	1.84 ± 0.02 ^d^	2.54 ± 0.06 ^a^	0.934 ± 0.07 ^a^
	0.05	3.14 ± 0.02 ^b^	3.37 ± 0.03 ^b^	1.20 ± 0.02 ^b^	2.17 ±0.02 ^c^	1.78 ± 0.03 ^b^	0.577 ± 0.09 ^b^
Tapioca	0.10	3.12 ± 0.04 ^b^	3.35 ± 0.04 ^bc^	1.07 ± 0.03 ^c^	2.28 ± 0.02 ^b^	1.60 ± 0.05 ^c^	0.527 ± 0.03 ^c^
	0.50	2.87 ± 0.09 ^c^	3.34 ± 0.03 ^c^	0.64 ± 0.02 ^d^	2.70 ±0.02 ^a^	0.99 ± 0.02 ^d^	0.356 ± 0.06 ^d^
	0.00	5.81 ± 0.01 ^a^	1.73 ± 0.03 ^a^	1.46 ± 0.01 ^a^	0.28 ± 0.02 ^d^	1.93 ± 0.02 ^a^	0.578 ± 0.01 ^a^
	0.05	3.21 ± 0.14 ^b^	1.72 ± 0.02 ^a^	1.28 ± 0.03 ^b^	0.44 ± 0.01 ^c^	1.87 ± 0.02 ^b^	0.597 ±0.01 ^a^
Corn	0.10	3.19 ± 0.01 ^b^	1.67 ± 0.03 ^b^	1.19 ± 0.03 ^c^	0.48 ± 0.02 ^b^	1.72 ± 0.07 ^c^	0.536 ± 0.06 ^b^
	0.50	2.82 ± 0.03 ^c^	1.65 ± 0.01 ^b^	0.96 ± 0.01 ^d^	0.70 ± 0.01 ^a^	1.52 ± 0.02 ^d^	0.548 ± 0.02 ^b^

* Values expressed are mean ± standard deviation (*n* = 3). Data of starch samples from the same botanical origin with different superscript letters in the same column were significantly different (*p* < 0.05). * PV = Peak viscosity; TV = Trough viscosity; BD = Breakdown viscosity (BD = FV – TV); FV = Final viscosity; SB = Setback viscosity (SB = FV − PV).

**Table 3:** Herschel–Bulkley model parameters.

Types of Starch	GDL Concentration (%)	Herschel–Bulkley Model Parameters *			
		Yield Stress (σ_ο_, Pa)	Consistency (*K,* Pa s*^n^*)	Flow Index (*n*)	R^2^
Potato starch	0	19.33 ± 0.10 ^a^	5.05 ± 0.04 ^a^	0.428 ± 0.003 ^a^	0.9937
	0.05	3.34 ± 1.84 ^b^	4.24 ± 0.12 ^b^	0.409 ± 0.009 ^b^	0.9976
	0.1	2.54 ±1.63 ^b^	4.23 ± 0.34 ^b^	0.412 ± 0.009 ^b^	0.9928
	0.5	2.60 ± 0.89 ^b^	3.83 ± 0.02 ^c^	0.402 ± 0.006 ^b^	0.9955
Tapioca starch	0	7.50 ± 0.53 ^a^	4.50 ± 0.34 ^a^	0.472 ± 0.019 ^b^	0.9900
	0.05	6.56 ± 0.12 ^a^	3.75 ± 0.27 ^b^	0.546 ± 0.015 ^a^	0.9977
	0.1	5.77 ± 2.75 ^a^	3.99 ± 0.30 ^b^	0.533 ± 0.014 ^a^	0.9906
	0.5	4.34 ± 0.41 ^a^	2.97 ± 0.12 ^c^	0.563 ± 0.009 ^a^	0.9967
Corn starch	0	36.33 ± 1.62 ^b^	3.49 ± 0.45 ^a^	0.448 ± 0.020 ^a^	0.9925
	0.05	35.99 ± 2.79 ^b^	3.49 ± 0.22 ^a^	0.352 ± 0.030 ^a^	0.9973
	0.1	41.81 ± 0.78 ^a^	3.49 ± 0.09 ^a^	0.453 ± 0.015 ^a^	0.9903
	0.5	41.98 ± 1.42 ^a^	3.22 ± 0.06 ^a^	0.459 ± 0.007 ^a^	0.9923

* Values expressed mean ± standard deviation (*n* = 3). Data of starch samples from the same botanical origin with different superscript letters in the same column were significantly different (*p* < 0.05).

## Data Availability

The data presented in this study are available on request from the corresponding author. The data are not publicly available due to privacy restrictions.
